# Therapeutic efficacy of equine botulism heptavalent antitoxin against all seven botulinum neurotoxins in symptomatic guinea pigs

**DOI:** 10.1371/journal.pone.0222670

**Published:** 2019-09-17

**Authors:** Douglas Barker, Karen T. Gillum, Nancy A. Niemuth, Shantha Kodihalli

**Affiliations:** 1 Research and Development, Emergent BioSolutions Canada Inc., Winnipeg, Manitoba, Canada; 2 Battelle Biomedical Research Center, West Jefferson, Columbus, Ohio, United States of America; CEA (Atomic and Alternative Energies Commission), FRANCE

## Abstract

Botulism neurotoxins are highly toxic and are potential agents for bioterrorism. The development of effective therapy is essential to counter the possible use of these toxins in military and bioterrorism scenarios, and to provide treatment in cases of natural intoxication. Guinea pigs were intoxicated with a lethal dose of botulinum neurotoxin serotypes A, B, C, D, E, F or G, and at onset of the clinical disease intoxicated animals were treated with either BAT^®^ [Botulism Antitoxin Heptavalent (A, B, C, D, E, F, G)–(Equine)] or placebo. BAT product treatment significantly (p<0.0001) enhanced survival compared to placebo for all botulinum neurotoxin serotypes and arrested or mitigated the progression of clinical signs of botulism intoxication. These results demonstrated the therapeutic efficacy of BAT product in guinea pigs and provided supporting evidence of effectiveness for licensure of BAT product under FDA 21 CFR Part 601 (Subpart H Animal Rule) as a therapeutic for botulism intoxication to serotypes A, B, C, D, E, F or G in adults and pediatric patients.

## Introduction

Botulinum neurotoxins (BoNTs) are considered to be some of the most toxic substances known, with an estimated human lethal dose fifty (HLD_50_) of 1 ng/kg body weight [1]. Produced from spore-forming Gram-positive bacteria belonging to the genus *Clostridium*, BoNTs cause paralysis by blocking the release of acetylcholine at peripheral cholinergic nerve terminals of the skeletal and autonomic nervous systems [2]. BoNTs have been classified as category A biothreat agents in the United States [3]. The rationale behind this designation is the extreme potency of the toxin, the relative ease with which it can be isolated and used with malice and the severity of the clinical disease caused by the toxin [4].

In the United States, a total of 182 confirmed and 13 probable cases of botulism were reported to the Centers for Disease Control and Prevention (CDC) in 2017 [5]. In Europe, 201 suspected and 146 confirmed cases were reported in 2015 by a total of 18 European Union/European Economic Area (EU/EEA) countries with a notification rate of <0.1 cases per 100,000 population. Human botulism mortality rates have been reported as high as 60% [6,7]; however, with improved supportive care including respiratory support and antitoxins, mortality rates have decreased significantly in recent years [5,8]. The duration of hospitalization and length of stay in the intensive care unit (ICU) continues to present a significant burden to the healthcare system.

Humans are susceptible to all seven serotypes; thus, any one of them could be used for bioterrorism [9–20]. However only certain serotypes are associated with human botulism, including BoNT serotypes A, B, E and F. There are currently no FDA approved vaccines available for prevention of botulism in humans against any of the seven serotypes, and until the licensure of BAT^®^ [Botulism Antitoxin Heptavalent (A, B, C, D, E, F, G)–(Equine)] in the United States, no therapeutic was available to treat intoxication by all seven BoNT serotypes. The approval of equine botulism antitoxin in the past was based on clinical experience; however, the botulism incidence is too low to conduct carefully controlled clinical trials. Therefore, BAT product was developed for licensure in the United States under 21 CFR Part 601 (Subpart H, Animal Rule), ‘Approval of Biological Products When Human Efficacy Studies Are Not Ethical or Feasible.’ Under this rule, approval is based on adequate and well-controlled animal efficacy studies in two animal models, to establish that the drug is reasonably likely to produce clinical benefit in humans, in addition to establishing safety in humans.

Furthermore, a clearly defined trigger for initiation of treatment is required for use in animal efficacy studies for the treatment indication. BAT product was licensed by the United States Food and Drug Administration on 22 March 2013. It is currently the only botulism antitoxin licensed for the treatment of symptomatic botulism following documented or suspected exposure to any of the known seven BoNT serotypes in adults and pediatric patients.

BAT product is a sterile solution of F(ab’)_2_ and F(ab')_2_-related antibody fragments prepared by blending plasma obtained from horses immunized with a specific BoNT serotype (A, B, C, D, E, F or G) of botulinum toxoid and toxin into a final heptavalent product. The guinea pig was selected as a relevant model for efficacy evaluation because of its susceptibility to all seven BoNTs [21]. In addition, there is a large body of data demonstrating the reproducibility and usefulness of guinea pigs for the efficacy evaluation of botulism vaccines and antitoxins [21–24]. Although the subtle signs of botulism such as ptosis are not visible in guinea pigs, intoxication results in muscular weakness, respiratory distress, paralysis and death mimicking the human clinical scenario [21]; thus, the response in these animals is predictive of the response in humans, an important consideration for product evaluation.

The therapeutic efficacy of BAT product in comparison to a placebo (46.6% vs.0%) against BoNT serotype A in rhesus macaques has been reported previously [25]. The post-exposure prophylactic efficacy of BAT product in comparison to the placebo against all seven serotypes (>95% survival vs.0% for each serotype) in guinea pigs has also been established [21]. The therapeutic efficacy of BAT product (i.e. when given after confirmed signs of intoxication) against any of the seven BoNT serotypes mimicking the clinical use of BAT product had not been demonstrated.

For the evaluation of therapeutic efficacy, the BAT product was administered to symptomatic guinea pigs to mimic the product use in a clinical setting. The results of these pivotal studies along with the demonstrated effectiveness of BAT product in rhesus macaques [25] provided evidence of effectiveness for successful licensure.

## Materials and methods

### Experimental plan

Three separate studies were conducted.

Study 1 was conducted to evaluate the efficacy of BAT product in rescuing animals intoxicated with 4x GPIMLD_50_ (guinea pig intramuscular lethal dose 50) of BoNT serotypes A, C, D or F. This study was conducted in two phases; Phase 1 examined BoNT serotypes A and F, and Phase 2 examined BoNT serotypes C and D. Between thirty-one to thirty-five Hartley Guinea Pigs approximately gender-balanced were randomly assigned to either BAT product or placebo-control treatment groups for each BoNT serotype.

Study 2 was conducted to determine an intoxication dose for each of BoNT serotypes A, B, C, D, E, F and G that would be highly lethal while providing an adequate window to allow for the rescue of animals in a pivotal efficacy study. Seventy Hartley Guinea Pigs were randomized into seven groups of 10 animals each. Groups were gender balanced. Six groups received a single intramuscular injection of either BoNT serotype A or BoNT serotype E toxin at a dose equivalent to 4.0x, 2.0x or 1.5x GPIMLD_50_. A seventh group received saline only and acted as concurrent controls.

Study 3 was the pivotal 21-day survival study to demonstrate the efficacy of BAT product in rescuing animals intoxicated with BoNT serotypes A, B, C, D, E, F or G. Four hundred and seventy-six Hartley Guinea Pigs were randomized into fourteen groups of 34 animals each. Groups were gender-balanced. Animals were assigned to either BAT product or placebo-control treatment groups for each BoNT serotype. Due to a large number of animals used in this study, the study was conducted in seven separate phases, one for each BoNT serotype.

### Animals, husbandry and veterinary care

All experiments were conducted with Hartley guinea pigs (*Cavia porcellus*) supplied by Charles River Laboratories (Kingston, NY and Raleigh, NC locations). Each animal was received from the supplier with a surgically implanted jugular vein catheter. Animals that were in good health, free of malformations, and exhibiting no signs of clinical disease were released from quarantine by the BBRC facility veterinarian. Animal husbandry was in accordance with the standards specified in the “Guide for the Care and Use of Laboratory Animals” [26].

Animals were individually housed in polycarbonate cages in stainless steel racks, equipped with automated watering systems maintained on 24-hour continuous room lighting to allow for clinical observations. The bedding material utilized was Sani-chips® hardwood heat-treated chips. Animals received both water and PMI Certified Guinea Pig Diet 5026 ad libitum. Housing room temperatures were maintained at 68 to 75°F, and relative humidities were 32 to 70% while study animals were present. To reduce stress on the animals and to provide optional shelter from continuous room lighting, each animal was provided with a tinted individual plexiglass “hut” within the cage. The huts were removed from cages after observation of the first severe clinical sign or if the shelter interfered with the animal’s mobility. Animals were identified by individual cage cards and ear tags.

Guinea pigs were randomized pre-study intoxication on Day -1 to the treatment or control group.

The dose of neurotoxin administered was verified at BBRC using a mouse potency assay in male CD-1 (ICR) mice according to procedures described by Cardella [22].

### Body weight

The specified pre-intoxication weight range for animals on all studies was 400.0 to 500.0 g. Botulinum neurotoxin serotype D animals on Study 3 weighed between 350.0 g and 525.0 g prior to intoxication since the majority of females available for this cohort were underweight. Body weights were measured at the following time points: Study Days -1, 7, 10, 14 and 21.

### Botulinum neurotoxin intramuscular intoxication

Botulinum neurotoxin serotypes A, B, C, D and E were produced at the University of Wisconsin; BoNT serotypes F and G were produced at Metabiologics, Inc. (Madison, Wisconsin). Potencies of all BoNT serotypes are given in S1 Table. Botulinum neurotoxin serotypes A, B, C, D, E and F were received as ammonium sulfate precipitates and were reconstituted in phosphate-buffered saline (PBS). Botulinum neurotoxin serotype G was received in PBS, pH 6.2 (ammonium sulfate was removed by the manufacturer; therefore, no reconstitution was required). All BoNTs used in this study were in the complex form [27] consisting of the toxin and non-toxin-associated proteins. The LD_50_ was established previously [21]. The toxin was administered as a single 0.1 mL intramuscular (IM) injection of a specific BoNT serotype (A to G at doses equivalent to 4x GPIMLD_50_ to 1.5x GPIMLD_50_, see S1 Table) into the muscles of the right hind leg. Toxin dose administered was verified by mouse potency assay.

### Test and placebo control article intravenous administration

A preliminary study (Study 1) was conducted to evaluate the efficacy of BAT product against a limited number of toxin serotypes. For this study guinea pigs were intoxicated with BoNT toxins (A, C, D or F) at 4.0x GPIMLD_50_ via the IM route. Animals were treated IV with a single dose of placebo or a single scaled human dose of BAT product based on previous studies [21,25]. Briefly, assuming the average human weight of 70 kg and BAT product dose of 1 vial/person, the dose volume/kg of one scaled human dose equals to 1/70 of a vial or 0.16 mL/kg based on the 11.17 mL fill volume for the lot of BAT product used for these studies. This is consistent with FDA guidance, which states that for biologicals with molecular weight >100 kDa, the dose should be normalized on a mg/kg basis [28]. The toxin neutralization capacity administered based on the label claim for the lot of BAT product used for these studies is given in S2 Table. The product was administered immediately after the first observed moderate/severe clinical sign (treatment trigger) of intoxication.

For Study 2, no test or control article was administered.

For Study 3, guinea pigs were intoxicated with BoNT toxins (A, B, C, D, E, F or G) at 1.5 x GPIMLD_50_ via the IM route. The trigger for treatment with a single dose of placebo or a single scaled human dose of BAT product was defined as the fourth consecutive occurrence of a moderate or severe clinical sign of intoxication. Within 45 minutes of the trigger for treatment, each animal was intravenously administered with either test or control article. The treatment was administered via the indwelling venous catheter. Catheter patency was confirmed by visualization of blood in the catheter lumen immediately prior to treatment.

The test article was Botulism Antitoxin Heptavalent (serotypes A, B, C, D, E, F and G)–(equine), Lot ♯2060401Y, manufactured by Emergent BioSolutions Canada Inc. (Winnipeg, Manitoba Canada). It is a sterile solution which should be stored at -15 to -25°C. The manufacturing process, label claims for potency and toxin neutralization capacity for this product are described in detail by Emanuel and Kodihalli [21,25]. The same lot of BAT product was used for both Study 1 and Study 3. Toxin potency for each serotype ranged from 1,229 U/vial (BoNT serotype G) to 10,690 U/vial (BoNT serotype E, see S2 Table).

The control article was Botulism Antitoxin–Placebo, Lot #10703480 from Emergent BioSolutions Canada Inc. (Winnipeg, Manitoba, Canada). Botulism Antitoxin Placebo (normal equine immune globulin) was manufactured using a procedure similar to the manufacture of BAT product described elsewhere [21]. Placebo had a protein concentration of 50 mg/mL and potency of < 0.38 Units/vial against all seven BoNT. This material is described as a clear to opalescent liquid essentially free of foreign particles in a 20 cc Type 1 glass container. The same lot of Botulism Antitoxin Placebo was used for both Study 1 and Study 3.

The test and control article dilution material was normal saline (0.9% sodium chloride USP lot #J8H009) manufactured by Baxter. It was stored at controlled room temperature per manufacturer’s specifications.

### Euthanasia criteria

Any animals meeting a criterion for euthanasia were pre-terminally euthanized. The three criteria were: (1) any animal having a 25% or greater weight loss (when compared to last pre-intoxication body weight) in conjunction with any concurrent severe sign of intoxication; (2) any animal that has two consecutive observations of total paralysis; and (3) any animal that did not meet either of the first two criteria but was judged to be moribund. Only the Study Director (or the Battelle staff veterinarian in consultation with a lead technician if Study Director was not available) determined if an animal was moribund.

Animals that required euthanasia were first administered 0.3 mL xylazine hydrochloride (20 mg/mL) and 0.4 mL ketamine hydrochloride (100 mg/mL) by IM injection and then administered a lethal dose of Fatal-Plus (a euthanasia agent containing pentobarbital).

### Clinical observations

#### Efficacy of BAT product in animals intoxicated with 4x GPIMLD50 of botulinum neurotoxin (study 1)

Observations were initiated 12 hours post intoxication and performed hourly until every animal received treatment. Following treatment of the last animal, and continuing through Day 7, observations were made once every 3 hours and from Day 8 to 21 twice daily at least 6 hours apart.

#### Determination of botulism neurotoxin intoxication dose to demonstrate efficacy (study 2)

Observations were made at 6 hours post-challenge for BoNT serotype E, and within 18 hours post-challenge for BoNT serotype A. Animals were observed frequently (hourly to once every 8 hours for BoNT serotype A, half-hourly to hourly for BoNT serotype E) until study termination (Day 14). Animals judged to be in poor and deteriorating condition were euthanized.

#### Pivotal therapeutic efficacy of BAT product in guinea pigs intoxicated with 1.5x GPIMLD50 of botulism neurotoxin (study 3)

A pilot study was conducted prior to the pivotal efficacy study with a toxin dose of 1.5x GPIMLD_50_ for a few of the serotypes in which animals reverted to being asymptomatic (data not shown) after the onset of moderate clinical signs including right hind limb weakness (treatment trigger). To avoid treating animals with transient clinical signs, an objective, unambiguous and reliable trigger for treatment consistent across all serotypes was determined to be observation of four consecutive signs of any moderate (salivation, lacrimation, weak limbs, right hind limb weakness, changes in breathing sounds or patterns) or severe signs (forced abdominal respirations, total paralysis), although not necessarily four consecutive observations of the same sign of intoxication, by trained personnel. To ensure that the clinical sign assessment was objective and reproducible, the personnel conducting clinical observations were required to pass a proficiency test prior to study start confirming their ability to identify symptoms in guinea pigs after intoxication.

Observations were initiated within 6 hours post-intoxication for BoNT serotypes C, E and F; and within 12 hours post-intoxication for BoNT serotypes A, B, D and G. Guinea pigs were monitored for signs of intoxication either hourly ± 15 minutes (BoNT serotypes A, B, C, D and G) or every half hour ± 15 minutes (BoNT serotypes E and F). As soon as each animal showed its fourth consecutive moderate/severe clinical sign (i.e. trigger) of botulism, it was treated within 45 minutes with either BAT product or placebo-control (as appropriate).

Animals were treated upon four consecutive observations of moderate or severe signs of botulinum intoxication to provide confidence that animals are showing the actual onset of clinical disease. The majority of animals were treated based on observation of right hind limb weakness (defined as the animal failing to exhibit a clutch response to a blunt object inserted across the rear leg claws) or change in breathing sounds or pattern (defined as change in breathing with audible sounds, excessive deep or shallow or irregular breathing).

Each animal was intravenously administered with BAT product or placebo control (1.0 mL per 500 g body weight) article via an indwelling venous catheter adjusted to the correct volume immediately prior to administration. Time of administration was recorded immediately post-dose. Following treatment of the final animal in each serotype, observations were reduced to every 3 hours until study Day 10, or later if there were no clinical signs, they were reduced to once every 6 hours and from Day 15 to twice daily (at least 6 hours apart) until study termination on Day 21.

### Data analysis

Statistical analyses were performed using Stata (version 11.1). Survival was the primary endpoint, secondary endpoints including the incidence of clinical signs, time to death and clinical severity scores were analyzed. These secondary endpoints provide additional evidence of the efficacy of BAT product.

As Study 3 was the pivotal study for licensure under the Animal Rule (21 CFR 601.90) [29], analyses were conducted in a manner similar to that done for clinical trials. Specifically, an intent-to-treat (ITT) analysis set for each serotype was used, consisting of only those animals that were intoxicated with botulinum neurotoxin and survived to receive the test or placebo control article as appropriate for the treatment group to which they were assigned. Two animals (one intoxicated with BoNT serotype C and one intoxicated with BoNT serotype D) which died whose preceding clinical course was not consistent with BoNT intoxication and progression were retained for the analysis as the cause of death could not be determined based on pathology in these animals.

For each treatment and placebo group, the survival rate at 14- or 21-days post-intoxication was calculated, along with an exact 95% confidence interval for the survival rate using the Clopper-Pearson method.

Two-tailed Fisher’s exact tests were used to determine if there was a statistically significant difference between survival rates for the BAT product treatment group and the placebo control group or each serotype. Kaplan-Meier curves along with log-rank tests were used to compare the time to death between the BAT product treatment group and the placebo control groups for each serotype. The median time to death was determined along with a two-sided 95% confidence interval for each group using the product-limit method.

The incidence of clinical signs was calculated, along with an exact 95% confidence interval, using the Clopper-Pearson method. Two-tailed Fisher’s exact tests were then used to compare the incidence of clinical signs between the BAT product treatment group and the placebo control groups for each serotype. Kaplan-Meier curves along with log-rank tests were used to compare the time to onset of clinical signs between the BAT product treatment group and the placebo control group for each serotype. The median time to onset of clinical signs was determined along with a two-sided 95% confidence interval for each group using the product-limit method. This analysis was performed for each clinical sign, and the grouped clinical signs, by serotype.

The assessment of clinical severity was calculated for each animal in the analysis set, wherein mild clinical signs (lethargy) were assigned a value of “1”, moderate signs (salivation, lacrimation, right hind limb weakness, weak limbs, change in breathing sounds or patterns) were assigned a value of “2”, severe signs (forced abdominal respirations, total paralysis) a value of “3”. For those animals which succumbed or were euthanized, a score of “20” was assigned for that time point and for all subsequent time points to end of the study. At each clinical observation time point, the clinical severity scores were calculated (cumulative for all the clinical signs observed at that time point for each animal) and averaged for each treatment group. For animals that survived to study end, the final sacrifice record was not used in the analysis.

### Ethics statement

The research was conducted in compliance with the Animal Welfare Act (AWA, 7 U.S.C. §2131, 2002, 2007 and 2008) and other federal statutes and regulations relating to animals and experiments involving animals and adhered to the principles stated in the Guide for the Care and Use of Laboratory Animals [26]. All animal procedures were conducted under protocols (843-G005630 964-G005630 1180-G005630) approved by the Institutional Animal Care and Use Committees (IACUC) of Battelle Biomedical Research Center (BBRC), in according with IACUC guidelines, https://www.nal.usda.gov/awic/institutional-animal-care-and-use-committees.

## Results

### Efficacy of BAT product in guinea pigs intoxicated with 4x GPIMLD_50_ of neurotoxin (study 1)

The *in vivo* therapeutic efficacy of BAT product was evaluated in groups of guinea pigs (n = 31 to 35/group) that were intoxicated intramuscularly (IM) with respective BoNT serotypes (A, C, D, F) at 4.0x guinea pig intramuscular lethal dose fifty (GPIMLD_50_)_._ Animals were treated intravenously (IV) with a single scaled human dose of BAT product or placebo immediately after the first observed moderate/severe clinical sign (treatment trigger) of intoxication. All placebo-treated animals died in all BoNT serotypes tested, confirming the lethality of the selected challenge dose. Five out of 35 guinea pigs treated with BAT product survived in BoNT serotype C group, and 2/31 survived in BoNT serotype F group (Table 1). There were no survivors in BoNT serotypes A (0/33) or BoNT serotype D groups (0/33). Survival observed with BAT product treatment compared to placebo was very low (0% - 14%); consequently, survival was not statistically different between the treatment and placebo groups for any of the four BoNT serotypes tested. All animals that died had clinical observations consistent with BoNT intoxication before death.

**Table 1:** Mortality by BoNT serotype and group of guinea pigs intoxicated with 4x GPIMLD_50_ BoNT and treated with placebo or 1x scaled human dose of BAT product.

Group	Number Animals Dead/Total Animals Treated (Percent)			
	Serotype A	Serotype C	Serotype D	Serotype F
1.0x BAT Product	33/33 (100)	30/35 (86)	31/31 (100)	29/31 (94)
Placebo	33/33 (100)	34/34 (100)	32/32 (100)	32/32 (100)

The mean and median times to death are given in Table 2. Median survival time was significantly longer for BAT product-treated groups compared with the placebo groups (p < 0.0001, Log-Rank Test) for BoNT serotypes A, C and D (63 vs 56, 107 vs 66 and 74 vs 59 hours respectively). No difference was noted for BoNT serotype F, where the median time to death for both groups was 41 hours. The time to onset of moderate clinical signs (i.e. trigger for treatment initiation) was consistent between BAT product and placebo control groups for all BoNT serotypes; however, there was a delay in time to onset of severe clinical signs (immediately preceding death) in treatment groups for three (A, C, D) of the four BoNT serotypes tested (Table 2).

**Table 2:** Kaplan-Meier time to onset of clinical signs, time to death and log-rank test comparisons between treated (1x scaled human dose BAT product) and placebo groups for animals challenged with BoNT serotypes A, C, D and F.

Serotype	Test Group	Mean Time to Onset in Hours		Time to Death in Hours		Log-Rank Test Time to Death Comparison (P-value)
		Moderate Signs (Range)	Severe Signs (Range)	Mean (Range)	Median (95% Confidence Interval)	
A	1.0x BAT product	35 (30, 44)	64 (34, 113)	68 (42, 131)	63 (59, 64)	<0.0001*
	Placebo	35 (29, 43)	49 (34, 62)	54 (40, 65)	56 (47, 59)	
C	1.0x BAT product	34 (20, 45)	97 (56, 125)	105 (63, 131)	107 (86, 131)	<0.0001*
	Placebo	35 (22, 48)	68 (49, 110)	73 (59, 112)	66 (66, 71)	
D	1.0x BAT product	29 (22, 32)	85 (44, 110)	86 (38, 131)	74 (70, 95)	<0.0001*
	Placebo	28 (21, 43)	63 (41, 73)	60 (47, 80)	59 (55, 62)	
F	1.0x BAT product	30 (23, 42)	46 (24, 94)	42 (28, 61)	41 (37, 47)	0.8827
	Placebo	31 (23, 42)	56 (24, 121)	47 (25, 129)	41 (37, 47)	

* Comparison significant at the 0.05 level of significance

The clinical progression was very rapid at 4x GPIMLD_50_ dose of botulinum toxin for all 4 BoNT serotypes tested. There was an overlap in the time to onset of moderate (treatment trigger) and severe signs (Table 2). The delay in treatment while waiting for the onset of signs together with rapid clinical course resulted in survival of only 0–14% of animals (depending on BoNT serotype), compared to 0% of survival in the placebo groups. The time between the onset of clinical signs (time of treatment) and death was rapid and insufficient for BAT product treatment to prevent mortality, despite the excess neutralization capacity available in the dose of BAT product administered [21] (S2 Table). Thus, the guinea pig model using an exposure dose of 4x GPIMLD_50_ is not appropriate for evaluation of the therapeutic efficacy of BAT product due to rapid progression of clinical disease at higher toxin dose. Consequently, it was necessary to extend the duration of clinical signs (time between treatment and death) by selecting a lower and more appropriate toxin dose to provide a greater opportunity for demonstration of the therapeutic efficacy of BAT product in this model.

### Determination of botulism neurotoxin intoxication dose to demonstrate efficacy (study 2)

An extensive time course and lethality evaluation of a range of toxin doses (4x, 2x, and 1.5x GPIMLD_50,_ n = 10) of BoNT serotypes A and E were conducted to establish the toxin dose that provided the longest clinical course while still resulting in mortality for use in the pivotal therapeutic efficacy study. The selected BoNT toxins represent typical (Serotype A) and fast-acting (Serotype E) toxins. All animals intoxicated with BoNT serotypes (A and E) died or were euthanized before study Day 7. The median times to onset of clinical signs at 2x and 4x GPIMLD_50_ were comparable for both BoNT serotypes; however, time to onset was longer at 1.5x GPIMLD_50_ for BoNT serotype A (Table 3).

**Table 3:** Kaplan-Meier median (95% confidence interval) for time to onset of clinical signs for animals challenged with BoNT serotypes A and E and control group.

Clinical Sign	Kaplan-Meier Median (95% Confidence Interval) Time to Onset of Clinical Signs (Hours) in Each Study Group						
	Serotype A			Serotype E			Control
	1.5x GPIMLD_50_	2x GPIMLD_50_	4x GPIMLD_50_	1.5x GPIMLD_50_	2x GPIMLD_50_	4x GPIMLD_50_	PBS
Lethargy^1^	74 (71, 81)	45 (45, 59)	31 (27, 42)	24 (21, 26)	22 (21, 28)	(17, —)	—
Salivation^2^	73 (67, 75)	55 (52, 71)	39 (36, 49)	50 (29, 50)	33 (33, —)	24 (19, 24)	—
Lacrimation^2^	109 (103, 127)	74 (67, 89)	51 (46, —)	72 (—)	51 (28, 51)	26 (21, 26)	—
Weakness of the Right Hind Limb Only^2^	41 (34, 46)	27 (27, 31)	24 (21, 25)	15 (13, 17)	16 (13, 18)	13 (12, 13)	—
Weak Limbs^2^	61 (52, 69)	45 (40, 48)	32 (27, 37)	21 (20, 24)	20 (17, 24)	15 (14, 17)	—
Noticeable Change in Breathing Sound Rate or Pattern^2^	48 (36, 48)	35 (33, 36)	29 (29, 32)	22 (21, 24)	19 (15, 21)	14 (13, 16)	—
Forced Abdominal Respirations^3^	153 (125, 165)	74 (69, 87)	43 (41, 45)	30 (26, 39)	26 (24, 30)	19 (17, 20)	—
Total Paralysis^3^	165 (165, —)	78 (69, 97)	45 (45, 47)	32 (30, 41)	29 (26, 34)	21 (20, 22)	—
Any Moderate Sign	37 (32, 42)	27 (27, 31)	24 (21, 25)	14 (10, 17)	16 (12, 18)	13 (12, 13)	—
Any Severe Sign	153 (125, 165)	72 (69, 87)	43 (41, 45)	30 (26, 39)	26 (24, 30)	19 (17, 20)	—
First Clinical Sign	37 (32, 42)	27 (27, 31)	24 (21, 25)	14 (10, 17)	16 (12, 18)	13 (12, 13)	—

— The clinical sign was not observed or the Kaplan-Meier estimates could not be calculated due to censoring

^1^ Mild signs of botulinum intoxication

^2^ Moderate signs of botulinum intoxication

^3^ Severe signs of botulinum intoxication

The duration of clinical signs decreased as the challenge dose increased for both BoNT serotypes. The median time from clinical onset to death in the 1.5x GPIMLD_50_ dose group was approximately 4.6x and 2.6x longer than that of the 4x GPIMLD_50_ dose group for BoNT serotypes A and E, respectively. The time to death of animals intoxicated with high toxin dose (4x GPIMLD_50_) was approximately 3.2x and 1.6x faster than the animals exposed to low dose (1.5x GPIMLD_50_) for BoNT serotypes A and E, respectively (Table 4).

**Table 4:** Summary of Kaplan-Meier median (95% confidence interval) duration of clinical signs (therapeutic window) and mean and median time to death for BoNT serotypes A and E.

Serotype	Group	Median Duration in Hours of Clinical Signs (Range)	Mean Time to Death in Hours (Range)	Median Time to Death in Hours (95% Confidence Interval)
A	1.5x GPIMLD_50_	121 (72, 129)	143 (102, 165)	165 (102, 165)
	2x GPIMLD_50_	63 (52, 71)	92 (69, 143)	92 (79, 99)
	4x GPIMLD_50_	26 (25, 34)	52 (46, 66)	51 (47, 57)
E	1.5x GPIMLD_50_	21 (12, 24)	39 (25, 95)	32 (30, 39)
	2x GPIMLD_50_	14 (11, 19)	31 (15, 57)	30 (28, 32)
	4x GPIMLD_50_	8 (7, 9)	21 (17, 28)	20 (20, 22)
Control	—	—	—	—

In general, animals challenged with BoNT serotype E had a shorter clinical course than those intoxicated with BoNT serotype A. A toxin dose of 1.5x GPIMLD_50_ was selected as the revised toxin dose for use in therapeutic efficacy studies for all seven toxin serotypes. This dose was expected to produce a more prolonged clinical course and consequently would provide an opportunity to demonstrate the therapeutic effect of BAT product while still resulting in complete mortality of control animals. Moderate clinical signs, including right hind limb weakness, were identified and selected as early signs for use as a trigger for treatment initiation.

### Pivotal therapeutic efficacy of BAT product in guinea pigs intoxicated with 1.5x GPIMLD_50_ of neurotoxin (study 3)

The pivotal study was a randomized, blinded, and controlled GLP study. A total of 616 guinea pigs were randomized to fourteen gender-balanced groups (n = 34) and were intoxicated with a dose equivalent to 1.5x GPIMLD_50_ of the appropriate BoNT serotype (serotype A, B, C, D, E, F or G) given as a single intramuscular (IM) injection to the right hind limb. Due to the large sample size, the study was conducted independently for each BoNT serotype. At four consecutive occurrences of any moderate or severe signs, animals were treated IV with one human-scaled dose of BAT product.

There was a statistically significant (Fisher’s Exact Test, p<0.0001) enhancement in survival achieved with 1x scaled human dose of BAT product when compared to placebo for all BoNT serotypes (Table 5). Most treated animals, along with all placebo control animals, continued to progress from the trigger (right hind limb weakness in most cases) to develop systemic clinical signs such as a change in breathing and weak limbs. The treatment with BAT product resulted in virtually complete survival irrespective of the intoxicating BoNT serotype; however, mortality was lower than expected among BoNT serotype G placebo group. Even with the lower rate of mortality in the placebo group, a statistically significant improvement in survival of guinea pigs exposed to BoNT serotype G was achieved with BAT product treatment.

**Table 5:** Summary of survival with fisher’s exact test comparisons and Kaplan-Meier median time to death with log-rank test comparisons between BAT product-treated (1x scaled human dose) and placebo control groups in guinea pigs intoxicated with 1.5x GPIMLD_50_ BoNT serotypes A, B, C, D, E, F and G.

BoNT Serotype	Group	Treatment Dose Level	Median Time to Treatment (Min, Max)^4^ in Hours	Survival (percent)	Two-Sided Fisher’s Exact Test Comparison (p-value)	Kaplan-Meier Median Time to Death (95% Confidence Interval) in Hours	Log-Rank Test Time-to-Death Comparison (p-value)
A	A1	1.0x BAT Product	17 (15, 23)	34/34 (100%)	<0.0001*	—(—)	<0.0001*
	A2	Placebo Control^2^	17 (16, 29)	0/34 (0%)		99 (87, 113)	
B	B1	1.0x BAT Product ^1^	26 (20, 29)	34/34 (100%)	<0.0001*	—(—)	<0.0001*
	B2	Placebo Control^2^	25 (19, 29)	1/34 (3%)		94 (94, 112)	
C	C1	1.0x BAT Product ^1^	22 (12, 26)	33/34 (97%)	<0.0001*	—(—)	<0.0001*
	C2	Placebo Control^2^	22 (12, 26)	4/34 (12%)		114 (111, 141)	
D	D1	1.0x BAT Product ^1^	24 (22, 37)	33/34 (97%)	<0.0001*	—(—)	<0.0001*
	D2	Placebo Control^2^	24 (22, 37)	5/34 (15%)		156 (141, 180)	
E	E1	1.0x BAT Product ^1^	9 (7, 16)	34/34 (100%)	<0.0001*	—(—)	<0.0001*
	E2	Placebo Control^2^	8 (8, 10)	0/34 (0%)		29 (27, 30)	
F	F1	1.0x BAT Product ^1^	15 (11, 20)	34/34 (100%)	<0.0001*	—(—)	<0.0001*
	F2	Placebo Control^2^	15 (10, 20)	4/34 (12%)		58 (45, 68)	
G	G1	1.0x BAT Product ^1^	23 (15, 28)	34/34 (100%)	<0.0001*	—(—)	<0.0001*
	G2	Placebo Control^2^	22 (16, 29)	17/34 (50%)		168 (143, —)^3^	

^1^ Compared to proposed human clinical BAT product dose (mL/kg basis)

^2^ Normal Equine Immune Globulin

^3^ The upper bound of the 95 percent confidence interval could not be estimated due to the high incidence of censoring

^4^ Treatment was triggered by four consecutive observations of moderate or severe signs of botulism intoxication;—Either animal death was not observed (groups A1, B1, E1, F1, and G1) or the Kaplan-Meier estimates could not be calculated due to censoring (groups C1 and D1)

* Comparison significant at the 0.05 level of significance.

Two BAT product-treated animals died before the study end at 21 days; one intoxicated with BoNT serotype C (died on day 14) and one intoxicated with BoNT serotype D (died on day 8). In both deaths, the preceding clinical course was not consistent with BoNT intoxication progression. The cause of death could not be determined based on pathology. All placebo control animals (if surviving to the first weighing at either day 7 post-intoxication or upon exhibiting poor and deteriorating condition) lost weight before death. All but eight BAT product-treated animals gained weight throughout the post-intoxication period. The difference in weight changes between the treatment groups supports an overall clinical benefit with BAT product treatment (S1 Fig).

The median time to death could not be estimated for many of the treated groups since no mortality observed for BoNT serotypes A, B, E, F and G and only one death each was noted for serotypes C and D (Table 5). The treated groups had a significantly (p<0.0001) longer time to death compared to placebo controls for all seven serotypes.

Treatment with BAT product substantially reduced the overall incidence of severe clinical signs compared to placebo (Table 6). Despite immediate intervention after the onset of clinical signs, almost all (~99%) BAT product-treated animals developed the clinical sign change in breathing rate/sound or pattern in addition to the right hind limb weakness (which was the treatment trigger in a majority of the cases) at rates consistent with the placebo group. However, there was a reduced incidence of other moderate clinical signs, including weak limbs, lacrimation and salivation with treatment groups compared to placebo groups. The overall incidence of weak limbs in treatment groups ranged between 8.8% (BoNT serotype D, 3/34) and 76.5% (BoNT serotype F, 26/34) compared to 100% (34/34) in each of the placebo groups. The incidence of lacrimation in placebo groups ranged between 8.8% (3/34, BoNT serotype A) and 50% (17/34, BoNT serotype D) compared to a single BAT product-treated animal (BoNT serotype G). Salivation in treated animals was observed with exposure to BoNT serotype F (17.6%, 6/34) and BoNT serotype G (11.8%, 4/34), but was observed for all BoNT serotypes in placebo groups with incidence rates of between 8.8% (3/34) for BoNT serotype E and 91.2% (31/34) for BoNT serotype G. Incidence of severe signs of botulism was also higher in the placebo groups compared to treatment groups. For example, between 17.6% (BoNT serotypes C and D, both 6/34) and 97.1% (BoNT serotype F, 33/34) of placebo group animals exhibited forced abdominal respirations compared to only two BAT product-treated animals (one BoNT serotype C and one BoNT serotype F animal). Similarly, between 20.6% (BoNT serotype G, 7/34) and 100% (BoNT serotypes A and E, both 34/34) of placebo-treated animals progressed to total paralysis (severe sign requiring euthanasia) compared to 0% (0/34 for each serotype) of animals in the treatment groups. These results are indicative of the continued progression of the disease from mild to severe in placebo groups compared to the rapid arrest and subsequent reversal of the progress of illness among treated animals (Table 6).

**Table 6:** Incidence of clinical signs in BAT product-treated (1x scaled human dose BAT product) and placebo control groups of guinea pigs intoxicated with 1.5x GPIMLD_50_ BoNT serotypes A, B, C, D, E, F and G.

Clinical Sign	Number of Animals Showing Each Clinical Sign per Group													
	Serotype A		Serotype B		Serotype C		Serotype D		Serotype E		Serotype F		Serotype G	
	A1^1^	A2^2^	B1^1^	B2^2^	C1^1^	C2^2^	D1^1^	D2^2^	E1^1^	E2^2^	F1^1^	F2^2^	G1^1^	G2^2^
Total Number of Animals	34	34	34	34	34	34	34	34	34	34	34	34	34	34
Lethargy^3^	0	0	0	0	1	0	0	0	0	0	0	0	1	0
Salivation^4^	0	22	0	24	0	25	0	29	0	3	6	24	4	31
Lacrimation^4^	0	3	0	11	0	12	0	17	0	5	0	5	1	15
Right Hind Limb Weakness^4^	34	34	34	34	34	34	34	34	34	34	34	34	34	34
Weak Limbs^4^	5	34	10	34	6	34	3	34	16	34	26	34	14	34
Change in Breathing Sounds or Pattern^4^	34	34	34	34	33	34	30	34	34	34	34	34	34	34
Forced Abdominal Respirations^5^	0	9	0	15	1	6	0	6	0	32	1	33	0	16
Total Paralysis^5^	0	34	0	31	0	26	0	25	0	34	0	21	0	7
Any Moderate Clinical Sign	34	34	34	34	34	34	34	34	34	34	34	34	34	34
Any Severe Clinical Sign	0	34	0	32	1	27	0	27	0	34	1	33	0	17
Any Clinical Sign	34	34	34	34	34	34	34	34	34	34	34	34	34	34

^1^ Group IDs ending in “1” are BAT product -treated

^2^ Group IDs ending in “2” are Placebo controls

^3^ Mild signs of botulinum intoxication

^4^ Moderate signs of botulinum intoxication

^5^ Severe signs of botulinum intoxication

**Fig 1:**
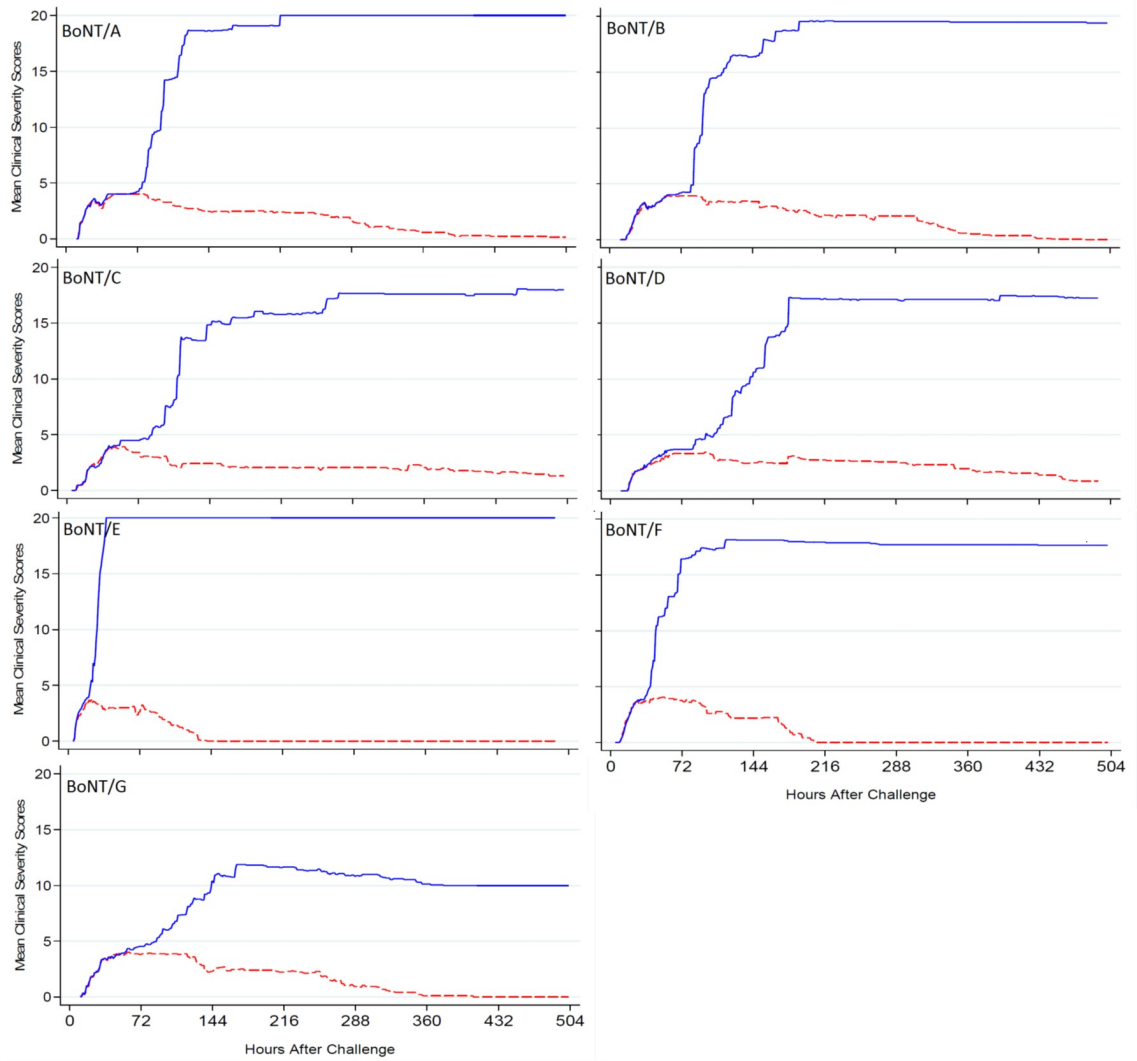
Mean clinical severity scores including scores for dead animals over time (Hours) by BoNT serotype and treatment group. Guinea pigs were intoxicated with 1.5x GPIMLD_50_ of BoNT serotypes A, B, C, D, E, F or G and subsequently treated with 1.0x BAT product (dashed red line) or placebo (solid blue line). Treatment was initiated after four consecutive observations of moderate or severe signs of botulinum intoxication. Animals were assigned a score of 1 (mild signs of intoxication), 2 (moderate signs of intoxication) or 3 (severe signs of intoxication) at each timepoint. A value of 20 was assigned for the time at which an animal succumbed or was euthanized, and for all subsequent time points to end of the study.

Severe signs (forced abdominal respiration and total paralysis) of botulism were observed almost exclusively in the placebo control animals, although the exact incidence varied with each BoNT serotype. There was no incidence of forced abdominal respiration in the treated group for BoNT serotype A. A significantly lower incidence of forced abdominal respiration in most BoNT serotypes (serotypes A, B, D, E, F and G) and total paralysis in all seven BoNT serotypes was observed in treated groups compared to placebo groups (p<0.05). Forced abdominal respiration was observed transiently (two consecutive half-hourly observations) for one animal intoxicated with BoNT serotype F and treated with BAT product. A second animal intoxicated with BoNT serotype C and treated with BAT product was found dead approximately three hours after first exhibiting forced abdominal respiration. Clinical progression was comparable between treatment and placebo groups but diverged approximately 21–58 hours post-treatment depending on the BoNT serotype (Fig 1). In general, the clinical severity scores demonstrate that for a period following intoxication and treatment, the clinical progression was comparable between BAT product and placebo groups; however, later, the clinical scores diverged. After this divergence the clinical severity score for BAT product-treated animals generally decreased as animals began to recover. In contrast, the clinical severity score for placebo-treated animals for most BoNT serotypes dramatically increased due to the onset of severe clinical signs or death. The clinical severity score continued to rise for placebo control animals until the end of the study or until all were dead or euthanized (Fig 1).

## Discussion

BAT product is an equine-derived heptavalent antitoxin licensed under the Animal Rule (21 CFR 601.90–95) for treatment of symptomatic botulism following documented or suspected exposure to BoNT serotypes A, B, C, D, E, F or G in adult and pediatric patients. The demonstrated efficacy of BAT product in rhesus macaques [25] along with the therapeutic effectiveness of BAT product against all seven BoNT serotypes in guinea pigs exhibiting clinical signs consistent with botulism provided the evidence of effectiveness in support of licensure under the Animal Rule in the US. This report for the first time demonstrates the therapeutic efficacy of a botulinum antitoxin against all seven BoNT serotypes in symptomatic guinea pigs.

Guinea pigs are susceptible to all seven BoNT serotypes [22,30,31]. Our detailed clinical course studies in guinea pigs confirmed the susceptibility to all seven serotypes [21]. While the primary disease of botulism (progressive paralysis resulting in death) is comparable between guinea pigs, rhesus macaques and humans, specific details such as the onset of clinical disease differ between the species [32].

The results of the studies described here demonstrate the effectiveness of BAT product against all seven BoNT serotypes when administered to systemically intoxicated guinea pigs after the onset of definitive clinical signs of botulism. Consistent with our previous studies in macaques [25], intervention with BAT product did not result in an immediate cessation of disease progression, likely due to a portion of the toxin having already entered neuronal cells where it is no longer accessible to the BAT product. The significant survival (nearly 100%) obtained in guinea pigs is due to the administration of BAT product as soon as possible following the onset of non-transient signs of intoxication in each animal. This finding is similar to previous reports of improved survival in humans with early treatment [33–36]. Although survival rates were significantly higher in BAT product-treated animals irrespective of BoNT serotype, the mortality rate in placebo controls was not universal. In particular, a significant proportion (50%) of placebo control animals intoxicated with BoNT serotype G survived to the end of the study. The lower than expected mortality was not believed to be due to an error in dosing as toxin dose formulation results confirmed the target dose (S3 Table). The lower than expected mortality is likely due to an inaccurate estimate of the LD_50_. This is critical as the 95% confidence intervals for the estimates of one GPIMLD_50_ for all seven BoNT serotypes are significant given the nature of the dose-response where a small increase in toxin dose is capable of changing survival rates from 0% to 100% [21] and is compounded by the relatively broad acceptance criteria associated with the toxin potency estimation due to the *in vivo* assay method used (S3 Table). Also, the actual dose delivered was 15% less than the target dose based on dose formulation analysis of challenge material. To address this uncertainty, the sample size determinations were made assuming survival rates of up to 65% for placebo-treated animals and not less than 95% for BAT product-treated animals.

Clinical severity scores are relevant for assessing the predictive efficacy of BAT product in human patients because of their comparability to the clinical scenario. In addition to survival benefit, the treatment also reduced the severity of the disease. Although intravenous administration of BAT product resulted in an immediate distribution within the circulatory system, the severity scores of treated animals were comparable to placebo controls until 2–3 days post-intoxication. The severity score for placebo control animals in most serotypes dramatically increased after that time resulting in death or euthanasia. In contrast, almost all treated animals (>98%) recovered completely by day 21. When observed as a cohesive whole, these data demonstrate the therapeutic efficacy of BAT product when given after the onset of systemic clinical disease.

These findings are consistent with the clinical experience, where administration of antitoxin did not result in immediate cessation in the clinical progression but did minimize the subsequent severity of the disease [35]. The duration of the recovery phase in human cases can range from several days to many months depending on the severity of the disease, serotype involved and time of treatment [10,34,37,38]. Depending on the severity, botulism intoxication can require extended periods of hospitalization and intensive care, which may not be feasible in a mass intoxication scenario [39]. Reducing the duration of hospital stays and the need for intensive care support with antitoxin treatment provide an opportunity for existing health care systems to continue to function in a mass exposure event scenario. While animal-derived anti-BoNT immunoglobulins can be immunogenic and may cause adverse events when administered to another species [39], no adverse events were noted in the animal studies presented. Overall, BAT product was well tolerated, consistent with the subsequently demonstrated favorable risk-benefit profile in patients with confirmed or suspected botulism treated with BAT product [36,40].

The significant protection obtained using the heptavalent antitoxin may be due to the polyclonal nature of the product that can target many different regions of the toxin and provide broader biological activity by interfering at various steps in the toxin pathway. Several monoclonal antibodies (mAbs) under development against BoNT toxin serotypes (A, B, E and F) have shown efficacy in animal models mostly in the form of a cocktail consisting of two or more mAbs to cover the breadth of response against each target toxin and counter naturally occurring toxin subtypes. The potential modification of toxin for use as a bioweapon limits the utility of mAbs as therapeutics [41–47]. Also, there are significant barriers to supply and cost with a monoclonal antibody treatment. Therefore, in a mass exposure event scenario, without knowing the exact serotype involved, a heptavalent product that can neutralize the entire spectrum to BoNT serotypes with a single dose is an effective countermeasure for bioterrorism concerns. For emergency preparedness and response, the United States government, through the Biomedical Advanced Research and Development Authority, has stockpiled BAT product in the Strategic National Stockpile.

The mechanism of action of BAT product is by the clearance of toxin in circulation and inhibiting the binding of the toxin to the neuronal cell surface receptor [48,49]. Published reports suggest that there is a correlation between the toxin dose and potential therapeutic window, and that antitoxin treatment is ineffective in experimental animals exposed to relatively high doses of BoNT [7,50]. Based on the standard neutralization capacity of one unit of toxin [21], and the large excess of antitoxin administered (relative to toxin exposure dose), the failure to rescue animals intoxicated with 4x GPIMLD_50_ of botulinum toxin in Study 1 can be attributed to the rapid progression of the disease due to the high intoxication dose of BoNT. This is evidenced by the overlap in times to onset of moderate and severe signs of botulism intoxication in the placebo groups intoxicated with BoNT serotypes A D and F. It is likely that neurons internalized lethal amounts of toxin before treatment. Thus, it was necessary to identify an intoxication dose that provided a wider window of opportunity for treatment, while still highly lethal to the control group. There was a clear relationship between clinical progression and the toxin dose in Study 2, with an adequate window of opportunity at lower but still highly lethal toxin challenge dose under experimental conditions. The data reported here show that a challenge dose of 1.5x GPIMLD_50_ of botulinum toxin is both relevant and reasonable for the evaluation of therapeutics against botulinum intoxication.

In conclusion, a single dose of BAT product administered to symptomatic guinea pigs following exposure to lethal quantities of BoNT (A, B, C, D, E, F or G) resulted in a statistically significant survival benefit compared to placebo control. Also, the progression of the clinical signs associated with botulinum intoxication was arrested with BAT product treatment. The results of these pivotal efficacy studies against all seven BoNT serotypes in guinea pig along with the efficacy against BoNT serotypes A in rhesus macaques [25] provided the evidence of effectiveness of BAT product in support of licensure under the Animal Rule in the US. Currently, BAT product is the only FDA approved treatment of symptomatic botulism following documented or suspected exposure to botulinum neurotoxin serotypes A, B, C, D, E, F or G in adult and pediatric patients.

## Supporting information

S1 Fig Observed percent body weight changes by serotype and treatment group on Day 7, 10, 14 and 21 post-intoxication. Guinea pigs were intoxicated with 1.5x GPIMLD_50_ of botulinum toxin serotypes A, B, C, D, E, F or G and subsequently treated with 1.0x BAT product (hollow blue circles) or placebo (hollow red triangles). Treatment was initiated after four consecutive observations of moderate or severe signs of botulinum intoxication with surviving animals being weighed at 7, 10, 14 and 21 days post-intoxication. Data points are for individual animals and show changes in weight as a percentage of baseline body weight.
(TIF)

S1 Table GPIMLD_50_ and Potency Values of BoNT Serotypes A, B, C, D, E, F, G.
(DOCX)

S2 Table Fold Excess Toxin Neutralization Capacity Provided by BAT Product When Administered as One Scaled Human Dose to Guinea Pigs Intoxicated with 4xGPIMLD_50_ of BoNT Serotypes A, B, C, D, E, F, G.
(DOCX)

S3 Table Summary of BoNT potency results per serotype and percent target between average and target potency values. (DOCX)
