# Supplementary figures and images for: Therapeutic efficacy of equine botulism heptavalent antitoxin against all seven botulinum neurotoxins in symptomatic guinea pigs
Source: PLoS One. 2019 Sep 17;14(9):e0222670. doi: 10.1371/journal.pone.0222670 (PMC6748678; doi:10.1371/journal.pone.0222670)

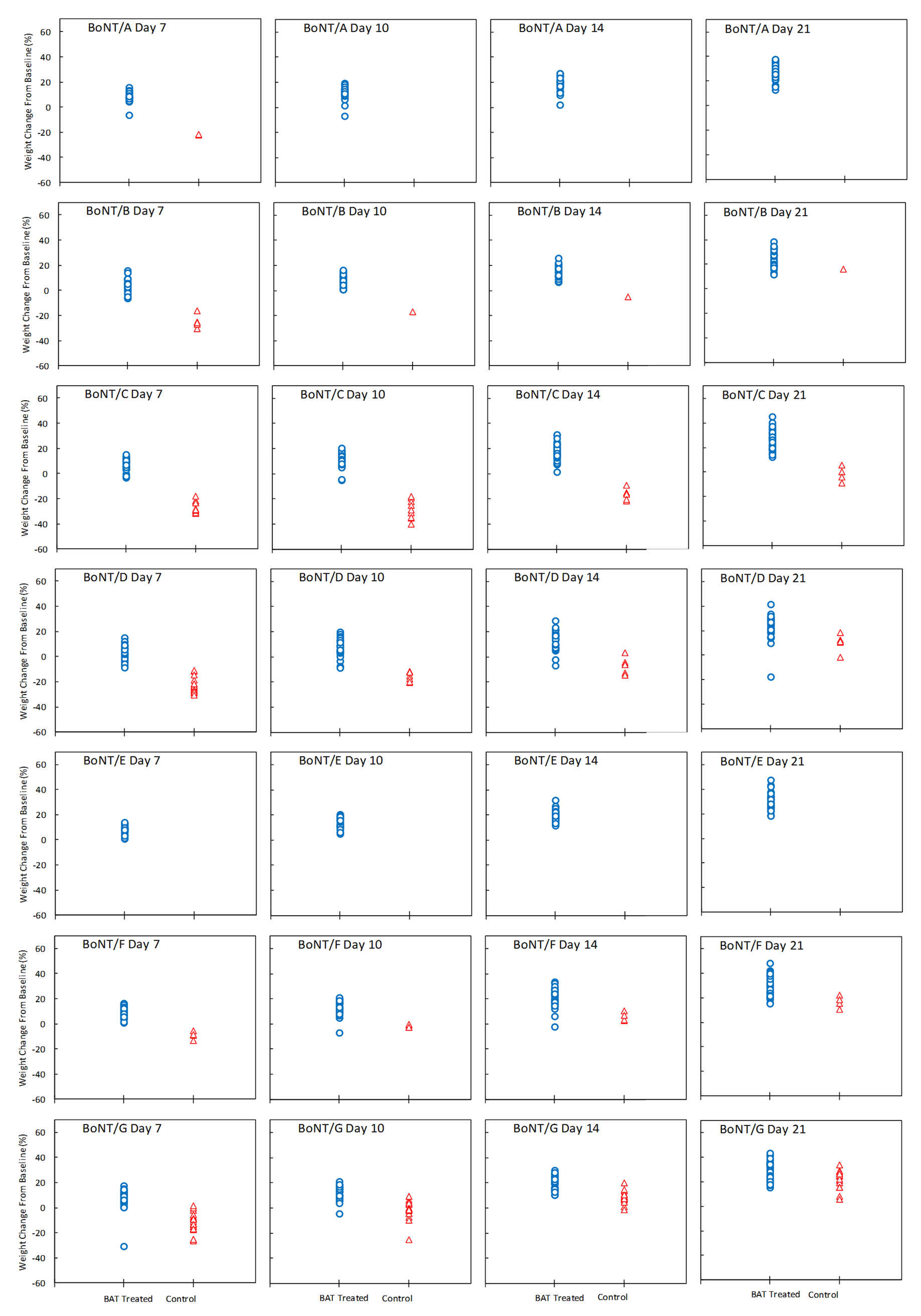

Supplement: S1 Fig — Guinea pigs were intoxicated with 1.5x GPIMLD50 of botulinum toxin serotypes A, B, C, D, E, F or G and subsequently treated with 1.0x BAT product (hollow blue circles) or placebo (hollow red triangles). Treatment was initiated after four consecutive observations of moderate or severe signs of botulinum intoxication with surviving animals being weighed at 7, 10, 14 and 21 days post-intoxication. Data points are for individual animals and show changes in weight as a percentage of baseline body weight. (TIF) [file pone.0222670.s001.tif]
