# Supplementary material for: Therapeutic efficacy of equine botulism heptavalent antitoxin against all seven botulinum neurotoxins in symptomatic guinea pigs
Source: PLoS One. 2019 Sep 17;14(9):e0222670. doi: 10.1371/journal.pone.0222670 (PMC6748678; doi:10.1371/journal.pone.0222670)
Supplement: S1 Table — GPIMLD50 and Potency Values of BoNT Serotypes A, B, C, D, E, F, G. (DOCX) [file pone.0222670.s002.docx]

**Table S1**: GPIMLD_50_ and Potency Values of BoNT Serotypes A, B, C, D, E, F, G

| **BoNT Serotype** | **Lot Number** | **Batch** | **1x GPIMLD_50_**  **(in MIPLD_50_ units)** | **1.5x GPIMLD_50_**  **(in MIPLD_50_ units)** | **4x GPIMLD_50_**  **(in MIPLD_50_ units)** | **Potency in MIPLD_50_ units**  **per mL (per μg)^1^** |
| --- | --- | --- | --- | --- | --- | --- |
| BoNT/A | A011995 | 2a | 4.5 | 6.8 | 18.0 | 172,559 (6,902.4) |
| BoNT/B | B011995 | 2a | 11.7 | 17.6 | 46.8 | 928,682 (18,209.5) |
| BoNT/C | C012495 | 2a | 2.6 | 3.9 | 101.4 | 12,009 (53.37) |
| BoNT/D | D022505 | 2a | 6.2 | 9.3 | 24.8 | 196,640 (1,080.4) |
| BoNT/E | E011295 | 2a | 81.2 | 121.8 | 324.8 | 333,122 (3,203.1) |
| BoNT/F | F033001-01 | 2a | 43.3 | 65.0 | 173.2 | 90,318 (1,557.2) |
| BoNT/G | G092905-01 | 4a | 56.4 | 84.6 | 225.6 | 399,056 (1,494.6) |

^1^Assumes protein concentrations as follows: BoNT/A: 25 μg/mL; BoNT/B: 51 μg/mL; BoNT/C: 225 μg/mL; BoNT/D: 182 μg/mL; BoNT/E: 104 μg/mL; BoNT/F: 58 μg/mL; BoNT/G: 267 μg/mL
