# Supplementary material for: Therapeutic efficacy of equine botulism heptavalent antitoxin against all seven botulinum neurotoxins in symptomatic guinea pigs
Source: PLoS One. 2019 Sep 17;14(9):e0222670. doi: 10.1371/journal.pone.0222670 (PMC6748678; doi:10.1371/journal.pone.0222670)
Supplement: S2 Table — Fold Excess Toxin Neutralization Capacity Provided by BAT Product When Administered as One Scaled Human Dose to Guinea Pigs Intoxicated with 4xGPIMLD50 of BoNT Serotypes A, B, C, D, E, F, G. (DOCX) [file pone.0222670.s003.docx]

**Table S2**: Fold Excess Toxin Neutralization Capacity Provided by BAT Product When Administered as One Scaled Human Dose to Guinea Pigs Intoxicated with 4xGPIMLD_50_ of BoNT Serotypes A, B, C, D, E, F, G

| BoNT Serotype | Label Claim U/vial (Target Potency U/vial; U/mg protein)^1^ | Antitoxin Potency Administered Based on Label Claim in U (U/kg)^2^ | Toxin Neutralization Capacity Administered Based on Label Claim (MIPLD_50_) | Toxin administered at 4xGPIMLD_50_ (MIPLD_50_) | Fold Excess Toxin Neutralization Capacity |
| --- | --- | --- | --- | --- | --- |
| A | >4500 (10399; 16.6) | >32 (>64) | 320000 | 18.0 | >17900x |
| B | >3300 (7071; 11.3) | >2 (>4 | 20000 | 46.8 | >500x |
| C | >3000 (7138; 11.4) | >21 (>42 | 210000 | 101.4 | >20000x |
| D | >600 (1452; 2.32) | >4 (>8 | 40000 | 24.8 | >1700x |
| E | >5100 (10690; 17.1) | >36 (>72 | 360000 | 324.8 | >1100x |
| F | >3000 (6546; 10.5) | >21 (>21 | 210000 | 173.2 | >1200x |
| G | >600 (1229; 1.96) | >4 (>8 | 40000 | 225.6 | >190x |

^1^Blended lot 2060401X fill volume 11.17 mL/vial and total protein per vial = 625.52 mg (56 mg/mL); ^2^Assumes 0.08 mL BAT product administered to a 500 g animal
