# Supplementary material for: Therapeutic efficacy of equine botulism heptavalent antitoxin against all seven botulinum neurotoxins in symptomatic guinea pigs
Source: PLoS One. 2019 Sep 17;14(9):e0222670. doi: 10.1371/journal.pone.0222670 (PMC6748678; doi:10.1371/journal.pone.0222670)
Supplement: S3 Table — (DOCX) [file pone.0222670.s004.docx]

**Table S3**: Summary of BoNT Potency Results per Serotype and Percent Target Between Average and Target Potency Values^1^

| **BoNT Serotype** | **Assay 1 (MIPLD50/mL)** | **Assay 2 (MIPLD50/mL)** | **Assay 3 (MIPLD50/mL)** | **Average (MIPLD50/mL) (A)** | **Target Intoxication Material Potency (MIPLD50/mL) (B)** | **Percent Target = (A/B)*100** |
| --- | --- | --- | --- | --- | --- | --- |
| BoNT/A | 69 | 86 | 68 | 74 | 68 | 109 |
| BoNT/B | 209 | 239 | 185 | 211 | 176 | 120 |
| BoNT/C | 40 | 41 | 35 | 39 | 39 | 100 |
| BoNT/D | 84 | 77 | 88 | 83 | 93 | 89 |
| BoNT/E | 1327 | 1450 | 1138 | 1305 | 1218 | 107 |
| BoNT/F | 459 | 546 | 546 | 517 | 650 | 80 |
| BoNT/G | 838 | 656 | 627 | 707 | 846 | 84 |

| ^1^ Acceptance criteria for BoNT averaged potency was ± 50% of the target dose. Assays were conducted using a mouse neutralization assay [21] |
| --- |
